# Prdx6 Plays a Main Role in the Crosstalk between Aging and Metabolic Sarcopenia

**DOI:** 10.3390/antiox9040329

**Published:** 2020-04-17

**Authors:** Francesca Pacifici, David Della-Morte, Francesca Piermarini, Roberto Arriga, Maria Giovanna Scioli, Barbara Capuani, Donatella Pastore, Andrea Coppola, Silvia Rea, Giulia Donadel, Aikaterini Andreadi, Pasquale Abete, Giuseppe Sconocchia, Alfonso Bellia, Augusto Orlandi, Davide Lauro

**Affiliations:** 1 Department of Systems Medicine, University of Rome “Tor Vergata”, 00133 Rome, Italy; pacifici.francesca@gmail.com (F.P.); david.dellamorte@uniroma2.it (D.D.-M.); francesca_piermarini@libero.it (F.P.); arrigaroby@libero.it (R.A.); barbara.capuani@uniroma2.it (B.C.); d.pastore3@inwind.it (D.P.); andreacopp@gmail.com (A.C.); siel-rea@hotmail.it (S.R.); andreadi@med.uniroma2.it (A.A.); bellia@med.uniroma2.it (A.B.); 2 Department of Human Sciences and Quality of Life Promotion, San Raffaele Roma Open University, 00166 Rome, Italy; 3 Department of Neurology and Evelyn F. McKnight Brain Institute, Miller School of Medicine, University of Miami, Miami, FL 33136, USA; 4 Department of Biomedicine and Prevention, Anatomic Pathology Section, University of Rome “Tor Vergata”, 00133 Rome, Italy; scioli@med.uniroma2.it (M.G.S.); orlandi@uniroma2.it (A.O.); 5 Department of Clinical Sciences and Translational Medicine, University of Rome Tor Vergata, 00133 Rome, Italy; donadel@uniroma2.it; 6 Department of Translational Medical Sciences, University of Naples “Federico II”, 80138 Naples, Italy; p.abete@unina.it; 7 Institute of Translational Pharmacology, National Research Council, 00133 Rome, Italy; giuseppe.sconocchia@cnr.it; 8 Department of Medical Sciences, Fondazione Policlinico Tor Vergata, 00133 Rome, Italy

**Keywords:** aging, insulin resistance, peroxiredoxin6, diabetes mellitus, sarcopenia, SIRT1

## Abstract

With the increase in average life expectancy, several individuals are affected by age-associated non-communicable chronic diseases (NCDs). The presence of NCDs, such as type 2 diabetes mellitus (T2DM), leads to the reduction in skeletal muscle mass, a pathological condition defined as sarcopenia. A key factor linking sarcopenia with cellular senescence and diabetes mellitus (DM) is oxidative stress. We previously reported as the absence of Peroxiredoxin 6 (Prdx6), an antioxidant enzyme implicated in maintaining intracellular redox homeostasis, induces an early-stage of T2DM. In the present study we sought to understand the role of Prdx6 in the crosstalk between aging and diabetic sarcopenia, by using Prdx6 knockout (*Prdx6^-/-^*) mice. Absence of Prdx6 reduced telomeres length and Sirtuin1 (SIRT1) nuclear localization. An increase in Sa-β-Gal activity and p53-p21 pro-aging pathway were also evident. An impairment in IGF-1 (Insulin-like Groth Factor-1)/Akt-1/mTOR pathway leading to a relative increase in Forkhead Box O1 (FOXO1) nuclear localization and in a decrease of muscle differentiation as per lower levels of myoblast determination protein 1 (MyoD) was observed. Muscle atrophy was also present in *Prdx6^-/-^* mice by the increase in Muscle RING finger 1 (MuRF1) levels and proteins ubiquitination associated to a reduction in muscle strength. The present study, innovatively, highlights a fundamental role of Prdx6, in the crosstalk between aging, sarcopenia, and DM.

## 1. Introduction

With the continuous increase in average life expectancy in western countries, we are witnessing a growing number of individuals affected by age-associated non-communicable chronic diseases (NCDs) [1]. The higher incidence of NCDs in elderlies increase exponentially the risk in losing muscle mass [2]. This pathological phenomenon, typical of aging, is defined as sarcopenia, which is represented by an impairment of muscle fibers regenerative power and by an altered differentiation of progenitor cells [3,4]. Sarcopenia, which affects elderly patients, is similar to the ones found in patients with chronic diseases [5]. However, the prevalence of muscle loss varies from around 60% in elderly patients with general chronic diseases to 90% in elderlies with metabolic disorders [5], suggesting a specific interplay between sarcopenia, aging, and metabolism that should be clarified.

Among the metabolic disorders associated with higher risk in sarcopenia type 2 diabetes mellitus (T2DM), is a multifactorial disease characterized mainly by hyperglycemia, insulin resistance, increase in oxidative stress, and low-grade inflammation [6]. The rise in oxidative stress is typical of T2DM and has been indicated as the key factor linking progression of cellular senescence, diabetes mellitus, and sarcopenia [7]. Intriguingly, in Peroxiredoxin 6 knockout (*Prdx6^-/-^*) mice model, we previously reported as the absence of Prdx6, induces a phenotype similar to early-stage of T2DM caused by both reduced glucose-stimulated insulin secretion and increased skeletal muscle insulin resistance with mild hyperglycemia [8]. Prdx6 is an antioxidant enzyme belonging to the peroxiredoxins family, with both peroxidase and phospholipase A2 activity implicated in maintaining intracellular redox homeostasis and lipid-glucose metabolism [9,10]. We also demonstrated as *Prdx6^-/-^* mice presented morphological and ultrastructural changes in islets of Langerhans and liver as well as altered inflammatory parameters [8], suggesting a pro-senescent phenotype. Accordingly, Prdx6 levels were inversely correlated with cellular aging; a decline of Prdx6 expression, indeed, was reported in aged ocular cells [11]. It is important to remark that the subcellular localization of Prdx6 is crucial for its enzymatic activities [12]. Prdx6 has higher phospholipase A2 activity at an acidic pH, and higher peroxidase activity at a neutral pH [12]. Moreover, Prdx6 from the cytosol and lysosomal-type organelles can move to the mitochondria and cellular membrane, by controlling cellular oxidative stress status [12]. All these Prdx6 functions may be influenced by cellular redox state that may change across aging and other metabolic variations.

However, these findings, suggest a key role of Prdx6 on the cellular oxidative stress mechanisms inducing aging-related disorders, such as T2DM and sarcopenia. So far, other markers, like telomeres shortening, and deregulation on Sirtuin 1 (SIRT1) pathway, have been already proposed for this process [13]. Aging has been further indicated as the most important risk factor for sarcopenia per se [14]. Moreover, oxidative stress may reduce skeletal muscle protein synthesis through alterations of insulin signal transduction [3], impairment of intracellular pathway regulating muscle cell differentiation [15], and activation of signaling cascade leading to muscle mass degradation [16].

However, so far, data on the direct association between Prdx6, aging, and its related diseases, such as diabetic sarcopenia, are sparse. Therefore, based on previous evidences, the main objective of the present study is to understand the role of this antioxidant enzyme in the crosstalk between diabetes, aging, and sarcopenia. A better knowledge on the role of this antioxidant enzyme could allow us to develop preventive strategies against metabolic dysfunctions, such as T2DM, and, consequently, on the related sarcopenia, especially in elderly patients. *Prdx6^-/-^* mice were used in order to establish a model of premature cellular aging, and to study the systemic effect of Prdx6 deletion avoiding a cell-specific phenomenon by using in vitro models.

## 2. Materials and Methods

### 2.1. Animal Models and Treatment

Wild type (wt) (C57BL/6J) mice were obtained from the Jackson Laboratory (Bar Harbor, ME, USA), while Prdx6 knockout (*Prdx6^-/-^*) (C57/BL6:129 SvJ) mice were gently given by Professor Xiaosong Wang [15]. All mice were maintained on a 12 h light: 12 h darkness cycle in a temperature-controlled room and given free access to food and water. Three-months old male mice fasted overnight were anesthetized with Avertin (Tribromoethanol, 5 mg/10 g), and insulin (1 U/kg body weight; Sigma Aldrich, Saint Louis, Missouri, USA) were injected through the portal vein. According to our previous published results [8], at 5 min (min) after injection, when we observed the activation of insulin signaling, mice were killed by cervical dislocation and gastrocnemius muscle was excised and frozen immediately in liquid nitrogen. Five mice per each group were used. All experimental procedures were in accordance with the Guide for the care and use of laboratory animals, Eighth edition (2011) and performed in accordance with institutional animal care guidelines at the Service Center for Inter-Station Animal Technology at University of Rome Tor Vergata (Rome, Italy) (ethical protocol code is790/2018-PR). None effect on aging and/or sarcopenia incidence have been reported among these two strains.

### 2.2. Cell Culture and Differentiation

Murine skeletal muscle cell line (C2C12) was purchased from American Type Culture Collection (Manassas, VA, USA). Cells were cultured in Dulbecco’s modified Eagle’s medium (DMEM)) supplemented with 20% fetal bovine serum (FBS) and 100 U/mL penicillin/streptomycin (Thermo Scientific, Waltham, MA, USA). Cells were maintained at 37 °C in humidified air containing 5% CO_2_. To induce C2C12 differentiation, cells were plated in growth medium, which was replaced by differentiation medium composed by DMEM supplemented with 2% heat-inactivated horse serum (Biowest, Nuaillé France) and 100 U/mL penicillin/streptomycin after 48 h and maintained for 3 days [16].

### 2.3. Stably Silenced Murine Myoblast C2C12 Cell Line

Knockdown cells were produced by transfecting Hek293T packaging cells with the lentiviral pLKO vector together with the Pax2 (pMDLg/p and pRSV-Rev plasmids) and ENV (VSV-G) plasmids [17]. The pLKO vector containing a shRNA for Prdx6 or for GFP (used as control), were purchased from Sigma Aldrich, Saint Louis, MO, USA. The lentiviral suspension was supplemented with polybrene (4 μg/mL, Sigma Aldrich, Saint Louis, MO, USA), and then, the viral supernatants were added to the target cells and incubated for 8–12 h. The infection was done twice to augment the efficiency of transduction. Then, infected cells were examined starting from 48 h after procedure.

### 2.4. RT^2^ Profiler PCR Senescence Array

The RT^2^ profiler PCR array (QIAGEN, Hilden, Germany) was used to assess mouse senescence genes expression of 84 selected genes. RT^2^ Profiler PCR Array results were analyzed with RT^2^ Profiler PCR Array data analysis software, on the QIAGEN Web site [18], which gives us the average 2^–∆∆CT^ for mean ± SD for all wt and *Prdx6^-/-^* groups. The system, also, automatically calculated the *p* value that we reported on the bar graphs.

### 2.5. DNA Isolation and Average Telomere Length Ratio

DNA isolation was performed by using DNA Purification System kit (Promega, Fitchburg, WI, USA) according to the manufacturer’s protocol. Total of 20 mg of tissue were digested overnight and subsequently, samples were transferred to minicolumns and centrifuged. Then, minicolumns were washed and DNA samples were eluted.

The average telomere length ratio (ATLR) was measured from total genomic DNA using qRT-PCR method as reported by Callicott et al. [19]. Telomeres primers and 36B4 gene (as internal control) were used and 36B4 gene was employed as a reference for standard curve construction (from 3.75 to 90 ng). Forward telomeres primer sequence used for telomere length was: 5′ CGG TTT GTT TGG GTT TGG GTT TGG GTT TGG GTT TGG GTT 3′. Reverse primer sequence was: 5′ GGC TTG CCT TAC CCT TAC CCT TAC CCT TAC CCT TAC CCT 3′. Forward and reverse primers sequences for 36B4 gene were respectively: 5′ ACT GGT CTA GGA CCC GAG AAG 3′ and 5′ TCA ATG GTG CCT CTG GAG ATT 3′. For telomere and 36B4 assays, 100 nM of both forward and reverse primers, 12.5 μL Syber Green PCR Master Mix (Applied Biosystems, Foster City, CA, USA) were used. Reaction conditions were initial denaturation step at 95 °C for 10 min, followed by 30 cycles of 95 °C for 15 s, and a 56 °C anneal-extend step for 1 min for data collection.

### 2.6. Senescence-Associated Beta-Galactosidase Activity

Senescence-associated beta-galactosidase (SA-β-gal) activity Fluorometric assay kit (Cell Biolabs, San Diego, CA, USA) was utilized following the manufacturer’s protocol. Fifteen mg of frozen muscle was homogenized using a Dounce homogenizer. Homogenates were maintained on ice and then, centrifuged. Supernatants were collected and total protein levels were determined by using Bradford assay (Bio-Rad Laboratories, MI, Italy). Total of 20 µg of total lysate were used and incubated for 2 h at room temperature. The reaction was stopped and fluorescence was read with a fluorescence plate reader at 360 nm (excitation)/465 nm (emission).

### 2.7. Nuclear and Cytoplasmic Fractionation

NE-PER nuclear and cytoplasmic extraction (Thermo Scientific, Waltham, MA, USA) was performed following the manufacturer’s protocol. Briefly, 15 mg of frozen muscle were homogenized using a Dounce homogenizer, maintained on ice, and then centrifuged. Supernatants, containing the cytoplasmic proteins were collected and stored at –80 °C. The pellet, containing nuclei, was washed twice with phosphate buffered saline (PBS), lysed on ice, and then centrifuged. Supernatants, containing nuclear proteins were collected. Antibody against SIRT1 (Abcam, Cambridge, UK), beta-actin and FOXO1 (Cell Signaling Technology, Danvers, MA, USA), and H3 histone (Santa Cruz Biotechnology, Santa Cruz, CA, USA) were used.

### 2.8. Western Blot

Skeletal muscle, excised as previously described, and cells pellet were homogenized with 1 mL of ice-cold buffer containing 20 mM Tris (pH 7.6) and 137 mM NaCl, 1.5% NP40, 1 mM MgCl_2_, 1 mM CaCl_2_, Glycerol 10%, 2 mM phenylmethylsulfonyl fluoride (PMSF), 1X phosphatase inhibitor cocktails 2 and 3 (Sigma Aldrich, Saint Louis, MO, USA), and 1X protease inhibitor cocktail tablets (Roche Diagnostics GmbH, Mannheim Germany). The homogenates were put on ice for 30 min, and then spun at 14,000 rpm for 30 min. Supernatants containing proteins were collected and stored at –80 °C before analysis and protein concentrations were determined using Bradford assay (Bio-Rad Laboratories, MI, Italy). Then, 40 µg of protein lysates were loaded on pre-cast 4–12% gels (Thermo Scientific, Waltham, MA, USA) separated by SDS-PAGE and transferred to nitrocellulose membranes using Trans Blot TurboTM Transfer System (Bio-Rad Laboratories, MI, Italy). The antigen–antibody complexes were next detected with enhanced chemiluminescence (ECL) reagent (GE Healthcare, Little Chalfont, UK) followed by exposure of the blot to X-ray film. Bands were quantified using Gel DocTM XR + with Image LabTM Software (Bio-Rad Laboratories, MI, Italy). Afterwards, total protein levels were assessed by immunoblotting using antibody direct against SIRT1 (1:1000, Abcam, Cambridge, UK), vinculin, Ubiquitin and p21 (1:200, Santa Cruz Biotechnology, Santa Cruz, CA, USA), p-Akt-1 S^473^, Ak-1t, p-mTOR, mTOR, p-p38, p-38, (1:1000, Cell Signaling Technology, Danvers, Massachusetts, USA), MyoD and MuRF1 (1:1000 and 1:10,000 respectively, Novus Biological, Centennial, CO, USA), α-tubulin (1:5000, Sigma Aldrich, Saint Louis, MO, USA).

### 2.9. p53 Immunoprecipitation Assay

An immunoprecipitation assay was performed as reported by Pacifici et al. [8]. Briefly, antibody against p53 (5 μg/mg) was rocked for 1 h at 4 °C with protein G (GE Healthcare, Little Chalfont, UK), and centrifuged. Later, 1 mg muscle lysate was added to the complex and was immunoprecipitated by rocking overnight at 4 °C. Immunoprecipitates were washed, resuspended in LDS sample buffer (Thermo Scientific, Waltham, MA, USA), loaded on precast 4–12% gels (Thermo Scientific, Waltham, MA, USA), separated by SDS-PAGE, and subjected to immunoblot analysis as reported above. Antibodies against acetyl-lysine (1:200, Santa Cruz Biotechnology, Santa Cruz, CA, USA) and p53 (1:1000, Abcam, Cambridge, UK) were used.

### 2.10. IGF-1 Sera Levels

Sera levels of IGF-1 were measured using mouse Magnetic Luminex^®^ Assay (R&D Systems, Minneapolis, MN, USA). Labospace S.r.l. (Milan, Italy), conducted the assay in service following the manufacturer’s protocol.

### 2.11. Malondialdehyde (MDA) Assay

MDA assay was performed in order to measure the lipid peroxidation as a marker of oxidative stress, by using commercially available OxiSelect™ MDA Adduct Competitive ELISA Kit (Cell Biolabs, Inc., San Diego, CA, USA) by following the manufacturer’s protocol. Briefly, samples were added on the MDA conjugate-coated wells for 10 min. Subsequently, samples were incubated with anti-MDA antibody for 1 h, at room temperature. Following three washes, the secondary antibody was added for 1 h. Then, samples were incubated with the substrate solution from 2 to 20 min. The reaction was stopped by using stop solution and the absorbance at 450 nm was read using a microplate reader. For each sample the absorbance was adjusted for mg of proteins loaded.

### 2.12. Total RNA Extraction and Gene Expression

Total RNA was isolated from skeletal muscle by using Trizol reagent (Thermo Scientific, Waltham, MA, USA). The high capacity cDNA Archive kit (Applied Biosystems, Foster City, CA, USA) was used to reverse transcribe 2.5 micrograms of total RNA into cDNA. ABI PRISM 7500 System and TaqMan reagents (Applied Biosystems, Foster City, CA, USA). Qualitative real time RT-PCR analysis was performed with an ABI PRISM 7500 System, applying TaqMan reagents (Applied Biosystems, Foster City, CA, USA). Each sample was amplified in duplicate using standard conditions, beta-actin was used to normalize the results. ∆∆CT method was utilized to calculate the relative expression, as previously reported [8].

### 2.13. Grip Strength Test

Muscle strength was tested with a grip strength meter (BioSeb, Vitrolles, France) in three rounds that were then averaged for each mouse [20]. Mice were held by the tail and lowered with both forepaws and hind paws, toward the horizontally positioned mesh grid [20]. All the measurements were recorded in grams.

### 2.14. Histology and Immunohistochemical Staining

Gastrocnemius muscles were collected and fixed with formalin solution neutral buffered 10%. After dehydration and paraffin embedding, tissue sections were cut (5 μm-thick). Slides, dewaxed and hydrated, were stained with hamatoxylin and eosin (H-E) or reacted with mouse monoclonal anti-fast myosin skeletal heavy chain (1:1000; ab51263, Abcam, Cambridge, UK) and anti-slow myosin skeletal heavy chain (1:250; ab11083). Slides were analyzed under a light microscope (Eclipse E600, Nikon, Tokyo, Japan) and images captured (magnification 20X) by a digital camera (DXM1200F, Nikon) connected to the ACT-1 software (Nikon). Then, the cross-sectional area (CSA) was measured using the ImageJ software 2018 (NIH, Bethesda, Maryland, USA), as reported [21].

### 2.15. Statistical Analysis

We analyzed data using GraphPad Prism 5 (La Jolla, CA, USA). All data were expressed as mean ± SEM, as indicated (*n* = 5 mice per group). Two-way analysis of variance (ANOVA) with Bonferroni post-hoc test or unpaired on-tailed Student’s test, when appropriate was determined for statistical analysis and significance, a value with *p* < 0.05 was considered statistically significant.

## 3. Results

### 3.1. Lacking of Prdx6 Induces a Premature Phenotype of Senescence

**Figure 1:**
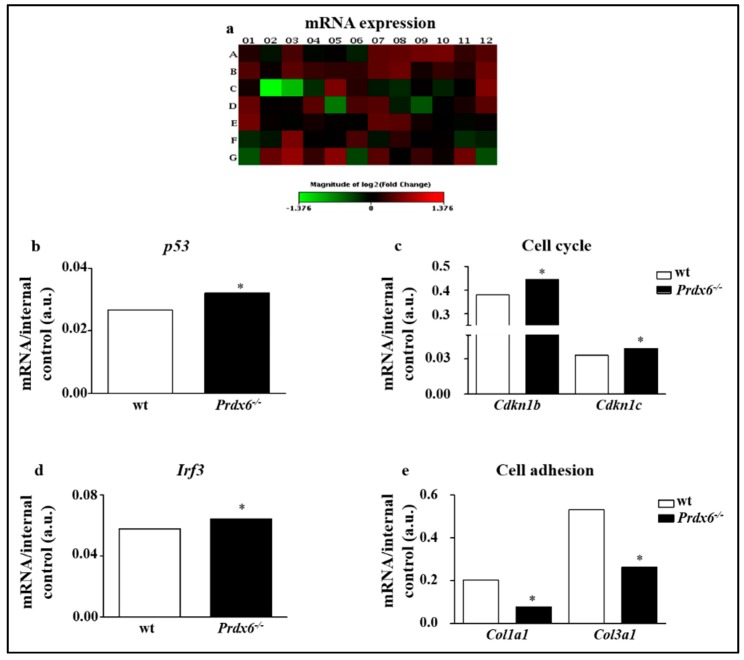
*Prdx6^-/-^* mice showed altered senescence genes profile. (**a**) Heatmap of expression changes in 84 key genes involved in the initiation and progression of cellular senescence in gastrocnemius skeletal muscle of *Prdx6^-/-^* mice. (**b**–**e**) Detailed expression levels of the indicated genes and pathways modulated in *Prdx6^-/-^* compared to control group. Bar graphs report the average mRNA levels reported as mean ± SD with the respective p-values automatically calculated by the QIAGEN program. All values were normalized with internal controls. * *p* < 0.05 (*n* = 5 mice per group).

In order to investigate the impact of Prdx6 on cellular aging, we screened the expression of 84 key genes involved in the senescence program on gastrocnemius of wt and *Prdx6^-/-^* mice (Figure 1a) (Table S1). A significant up-regulation of p53 (*p* < 0.05) (Figure 1b), cyclin-dependent kinase inhibitors b (Cdkn1b) (*p* < 0.05) and Cdkn1c (*p* < 0.05), was found in *Prdx6^-/-^* mice (Figure 1c). Moreover, interferon regulatory factor 3 (IRF3) was significantly enhanced in *Prdx6^-/-^* mice (*p* < 0.05) (Figure 1d). Conversely, a significant reduction of collagen type I alpha 1 (Col1a1) and collagen type III alpha 1 (Col3a1) expression was observed in *Prdx6^-/-^* mice (*p* < 0.005) (Figure 1e), suggesting as morphological and structural muscular changes typical of senescent phenotypes may occur in absence of Prdx6.

**Figure 2:**
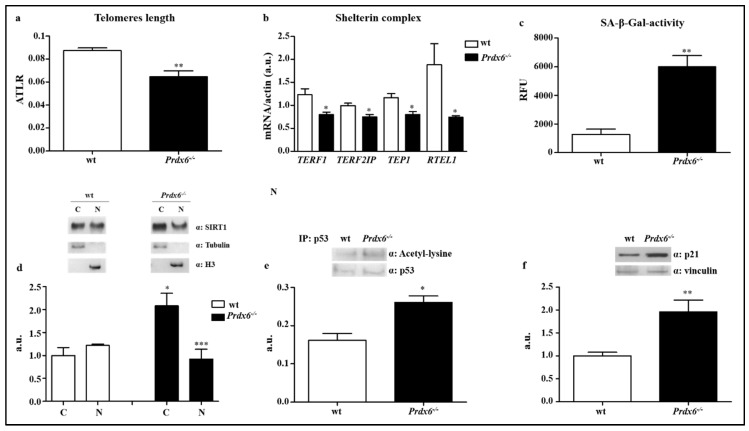
Prdx6 is crucial for SIRT1 nuclear translocation and p53/p21 mediated cell cycle survival. (**a**) The average telomere length ratio (ATLR) from wt (white bar) and *Prdx6^-/-^* mice (black bar), were evaluated. (**b**) Gene expression levels of the main factors involved in telomere stabilization and elongation were analyzed in fasted (overnight) wt (white bars) and *Prdx6^-/-^* (black bars) mice. (**c**) Senescence-associated β galactosidase activity (SA-β-gal) was assessed. (**d**) SIRT1 nuclear and cytoplasmic localization was quantified following cellular fractionation. * *p* < 0.05 *Prdx6^-/-^* vs. wt cytoplasm; *** *p* < 0.0005 *Prdx6^-/-^* vs. wt nucleus. (**e**) Immunoprecipitation assay for p53 was performed on gastrocnemius muscle of both mouse models. (**f**) p21 levels were enhanced in *Prdx6^-/-^* than wt mice. Data are reported as mean ± SE. * *p* < 0.05, ** *p* < 0.005 using Student t test. Graphs illustrate one of three separate studies, all yielding similar results (*n* = 5 mice per group). RFU = relative fluorescence unit; a.u., arbitrary units; C = cytoplasm; N = nucleus.

Then, in gastrocnemius, we assessed the average telomere length ratio (ATLR), which was significantly reduced in *Prdx6^-/-^* mice compared to wt (*p* < 0.005) (Figure 2a). Furthermore, we evaluated the effect of Prdx6 in the telomeric machinery by analyzing the expression levels of the most important genes belonging to the shelterin complex, which controls telomeres length. Specifically, as reported in Figure 2b, the expression levels of telomeric repeat-binding factor 1 (TERF1), telomeric repeat-binding factor 2-interacting protein 1 (TERF2IP), telomerase-associated protein 1 (TEP1), and regulator of telomere length 1 (RTEL1), were significantly reduced in *Prdx6^-/-^* mice compared to wt mice (*p* < 0.05).

To further confirm the effect of Prdx6 deletion on cellular aging processes, we also measured the senescence-associated β-galactosidase (SA-β-Gal) activity. *Prdx6^-/-^* mice showed increase in activity of SA-β-Gal compared to wt mice (*p* < 0.005) (Figure 2c), indicating an irreversible arrest of muscle cells growth linked to the activation of cellular aging processes.

### 3.2. Lacking in Prdx6 Impairs SIRT1 Nuclear Translocation Resulting in p53/p21 Pro-Aging Pathway Activation

Similarly to Prdx6, SIRT1 has shown to modulate insulin release and to be implicated in metabolic disorders [22]. Therefore, we examined the possible link between Prdx6 and SIRT1 in *Prdx6^-/-^* mice model, by analyzing SIRT1 subcellular localization in gastrocnemius. It is well established, indeed, that active SIRT1 translocates from cytoplasm into the nucleus. Accordingly, to pro-senescent phenotype, we expected to find a reduced nuclear localization of SIRT1 in *Prdx6^-/-^* mice. Following the nuclear and cytoplasmic proteins fractionation, indeed, we found that in *Prdx6^-/-^* mice SIRT1 localized predominantly in the cytoplasm (*p* < 0.05) rather than in the nucleus (*p* < 0.0005) compared to wt (Figure 2d), suggesting reduced levels of Prdx6 lead to an impairment of SIRT1 nuclear-cytoplasmic shuttling.

Previous evidences from the RT^2^ profiler array demonstrated that p53 gene expression was significantly enhanced in *Prdx6^-/-^* mice muscle (Figure 1b). To further validate a reduction in SIRT1 activity, we evaluated the levels of p53 acetylation, a SIRT1 non-histonic target. As expected, p53 acetylation was significantly increased in *Prdx6^-/-^* mice compared to wt mice (*p* < 0.05) (Figure 2e), confirming the SIRT1 reduced nuclear activity. Acetylated p53 induces p21, a cyclin-dependent kinase inhibitor 1 able to regulate cell cycle and directly blunt telomerase activity [23]. Consistently with the reduction of ATLR and increase in p53 activation, we observed a significantly enhanced steady-state levels of p21 in *Prdx6^-/-^* mice (*p* < 0.005) (Figure 2f). All these data indicate that deletion of Prdx6, by altering SIRT1 nuclear translocation, promotes the activation of the pro-aging p53-p21 pathway.

### 3.3. Prdx6 Deletion Induces Phenotype of Sarcopenia via IGF-1/Akt-1/FOXO1 Pathway

**Figure 3:**
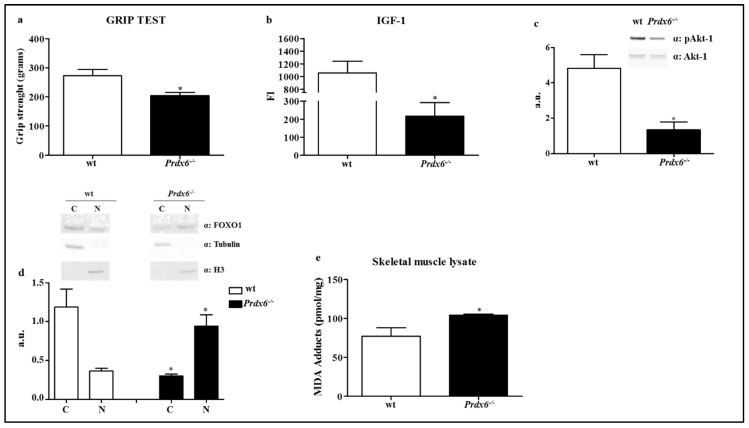
Absence of Prdx6 altered the IGF-1/Akt-1/FOXO1 pathway inducing Sarcopenia. (**a**) Mice muscle strength was measured by performing grip test in wt (white bar) and *Prdx6^-/-^* mice (black bar). (**b**) Sera levels of IGF-1 were evaluated. (**c**) Akt-1 phosphorylated (pAkt-1) levels at Ser^473^ were evaluated in gastrocnemius muscle lysate of *Prdx6^-/-^* and wt mice. (**d**) Subcellular localization of FOXO1 was analyzed in skeletal muscle of wt and *Prdx6^-/-^* mice. * *p* < 0.05 *Prdx6^-/-^* versus wt mice. (**e**) MDA adducts were in gastrocnemius muscle lysate of *Prdx6^-/-^* and wt mice. Data are expressed as mean ± SE. * *p* < 0.05 using Student *t* test. Graphs illustrate one of three separate studies, all yielding similar results (*n* = 5 mice per group). a.u. = arbitrary units; FI = fluorescence intensity; C = cytoplasm; N = nucleus.

Based on these data and on the previous findings, we aim to evaluate the presence of sarcopenia, as advanced phenotype of cellular aging, in Prdx6^-/-^ mouse model. In order to measure mice muscle strength, as marker of sarcopenia, we performed the grip test on both forelimbs and hindlimbs in wt and *Prdx6^-/-^* mice. As shown in Figure 3a, *Prdx6^-/-^* mice exerted lower force on mesh grid compared to wt mice (*p* < 0.05), suggesting a significant impairment of body whole muscle function related to Prdx6 loss.

Insulin-like growth factor-1 (IGF-1) was reported to be linked either with sarcopenia, or cellular senescence via SIRT1-p53 pathway [24]. Therefore, we measured sera levels of IGF-1, which were significantly reduced in *Prdx6^-/-^* mice compared to wt (*p* < 0.05) (Figure 3b). To further confirm the defect of IGF-1 signaling, we analyzed the phosphorylation of Akt-1 at Ser^473^, which represents the fully activated form of Akt-1 able to modulate several downstream targets, such as the intracellular localization of Forkhead Box O1 (FOXO1). According to the decrease in IGF-1 levels, *Prdx6^-/-^* mice exhibited reduced Akt-1phosphorylation levels compared to control group (*p* < 0.05) (Figure 3c).

FOXO1 plays a pivotal role in skeletal muscle homeostasis [25], and when phosphorylated by the fully active form of Akt-1, is inhibited and localized primarily in the cytoplasm. Therefore, we performed a subcellular fractionation to investigate the intracellular distribution of FOXO1. In Figure 3d, it is reported as in *Prdx6^-/-^* mice FOXO1 localized mainly in the nucleus, conversely to control mice (*p* < 0.05), and therefore, was significantly lowered in the cytoplasm (*p* < 0.05), confirming the impairment of IGF-1/Akt-1/FOXO1 pathway. The increase in FOXO1 nuclear localization may be also associated with c-Jun N-terminal kinase (JNK) activation, which is triggered by oxidative stress [26]. Accordingly, we observed both enhanced levels of oxidative stress in skeletal muscle of *Prdx6^-/-^* mice (Figure 3e), and increased activation on JNK as reported by Pacifici et al. [8]. Differently, no significant differences were observed in phosphorylated and total extracellular signal-regulated kinase (ERK1/2) levels (data not shown).

**Figure 4:**
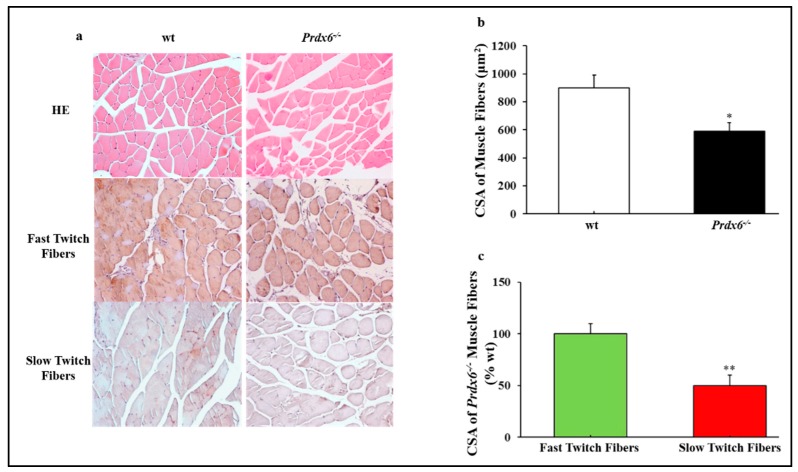
Histology evidences skeletal muscle atrophy in *Prdx6^-/-^* mice. **(a)** HE and immunohistochemical staining for fast and slow myosin heavy chain (magnification 20×). **(b)** CSA of muscle fibers (μm^2^). **(c)** CSA of *Prdx6^-/-^* fast and slow muscle fibers expressed as percentage of wt. * *p* < 0.05 and ** *p* < 0.01 *Prdx6^-/-^* vs wt. (*n* = 5 mice per group). HE = Hematoxilin-Eosin; CSA = cross-sectional area.

In order to confirm the presence of skeletal muscle atrophy in absence of Prdx6, typical of sarcopenia, we compared the histological features of wt and *Prdx6^-/-^* gastrocnemius muscles. Hematoxylin-eosin staining showed significant variations in size and organization in *Prdx6^-/-^* skeletal muscle fibers as demonstrated by the reduction in cross-sectional area (CSA) of muscle fibers in *Prdx6^-/-^* mice compared with wt (*p* < 0.05) (Figure 4a,b). In particular, immunohistochemical analysis revealed that CSA of slow muscle fibers in *Prdx6^-/-^* mice was lower compared to wt (*p* < 0.01), whereas CSA of fast muscle fibers did not change between the two groups (Figure 4c).

### 3.4. Loss of Prdx6 Blunts Muscle Differentiation and Proteins Synthesis Leading to Sarcopenia

**Figure 5:**
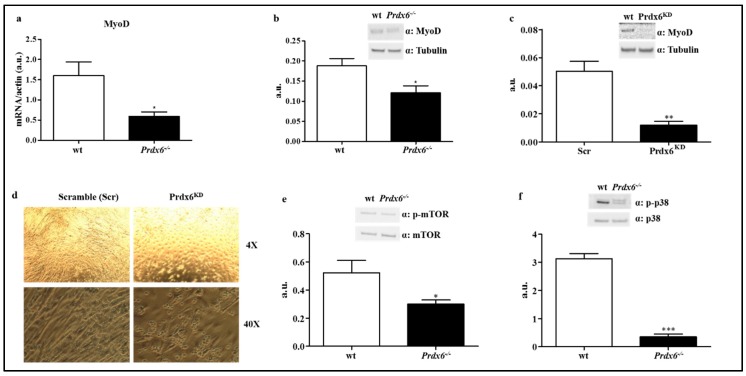
Suppression of Prdx6-reduced MyoD-mediated muscle differentiation. (**a**) MyoD gene expression was evaluated in gastrocnemius of wt (white bar) and *Prdx6^-/-^* (black bar) mice. (**b,c**) MyoD gene and protein levels were tested in murine myoblasts cells (C2C12) stably silenced for Prdx6 (Prdx6^KD^) (white bar) and in control cells (Scramble, Scr) (black bar). (**d**) Murine myoblasts cells differentiation at both 4× and 40× of magnification. (**e**) Protein phosphorylation levels of mTOR (p-mTOR) in gastrocnemius of *Prdx6^-/-^* and wt mice were analyzed. (**f**) Phosphorylation levels of p38 (p-p38) were measured in muscle of *Prdx6^-/-^* and wt mice. Results are expressed as mean ± SE. * *p* < 0.05, ** *p* < 0.005; *** *p* < 0.0005 using Student *t* test. Graphs illustrate one of three separate studies, all yielding similar results (*n* = 5 mice per group). a.u. = arbitrary units.

Muscle mass homeostasis is also significantly dependent on the differentiation of progenitor muscle cells [27]. A relevant role in the early stage of muscle differentiation is played by the myogenic regulatory factor myoblast determination protein 1 (MyoD), in which genetic expression is negatively regulated by nuclear FOXO1 [28]. Thus, according to our previous data, gene and protein expressions of MyoD in muscle of wt and *Prdx6^-/-^* mice were analyzed. We found that *Prdx6^-/-^* mice displayed significant diminished levels of both gene and protein, compared to wt mice (*p* < 0.05) (Figure 5a,b), in agreement with increased nuclear FOXO1 localization and activation.

Then, we validated the direct role of Prdx6 in regulating MyoD expression by testing MyoD levels in a murine myoblast cell line C2C12 stably silenced for Prdx6 (Prdx6^KD^). As reported in Figure 5c the absence of Prdx6 dramatically blunted MyoD protein levels compared to control scramble (Scr) cells (*p* < 0.005). Moreover, following myoblast differentiation, we reported as Prdx6^KD^ cells were not able to fully differentiate in myotubes compared to Scr cells, which, instead, assumed the elongated typical shape of mature myotubes (Figure 5d).

MyoD is activated by mTOR (mammalian target of rapamycin), which controls the anabolic and catabolic signaling of skeletal muscle mass, resulting in the modulation of muscle hypertrophy and sarcopenia. To be active as kinase, mTOR needs to be phosphorylated by Akt-1 at Ser^2448^ [29]. According to our data on MyoD, phosphorylated levels of mTOR were significantly reduced in *Prdx6^-/-^* compared to wt mice (*p* < 0.05) (Figure 5e), highlighting as in absence of Prdx6, mechanisms regulating muscle homeostasis are impaired. Further evidences are reported in Figure 5f, where it is shown as a significant reduction of phosphorylated p-38 in *Prdx6^-/-^* compared to wt mice (*p* < 0.0005). The mitogen-activated protein kinase (MAPK) p-38, when phosphorylated/activated, leads to muscle differentiation via MyoD activation [30].

### 3.5. Loss of Prdx6 Promotes Muscle Atrophy via MuRF1-Ubiquitin Pathway

**Figure 6:**
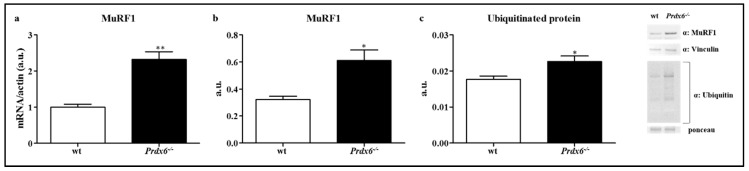
Prdx6 deletion activates MuRF1-Ubiquitin pathway. Gene (**a**) and protein (**b**) levels of MuRF1 were evaluated in gastrocnemius of wt (white bar) and *Prdx6^-/-^* (black bar) mice. (**c**) Ubiquitination of muscle proteins was analyzed in *Prdx6^-/-^* mice. Results are expressed as mean ± SE. * *p* < 0.05, ** *p* < 0.005 using Student *t* test. Graphs illustrate one of three separate studies, all yielding similar results (*n* = 5 mice per group). a.u. = arbitrary units.

Then, the main mechanism underlay muscle atrophy was also investigated, which begins when the regular turnover of the contractile proteins and organelles are impaired, especially for an increase in proteins ubiquitination. One of the most important E3 ubiquitin ligases expressed in skeletal muscle is Muscle RING finger 1 (MuRF1) that leads to polyubiquitination of proteins and then, to proteolysis [31], which is positively regulated by nuclear FOXO1 [28]. Therefore, we evaluated both gene and protein expression of MuRF1 in muscle of wt and *Prdx6^-/-^* mice. According to our findings, the mRNA levels of MuRF1 significantly increased in *Prdx6^-/-^* compared to wt mice (*p* < 0.005) (Figure 6a). Similar results were reported for MuRF1 protein (*p* < 0.05) (Figure 6b). Once assessed MuRF1 activation, we measured the ubiquitination levels of muscle proteins in both *Prdx6^-/-^* and wt mice. As expected, the levels of ubiquitinated proteins were higher in *Prdx6^-/-^* (*p* < 0.05) (Figure 6c), suggesting that even the mechanism of muscle atrophy is implicated in Prdx6-associated sarcopenia.

## 4. Discussion

In the present study, we reported as deletion of the antioxidant enzyme Prdx6 accelerates cellular aging phenotypes, since *Prdx6^-/-^* mice showed a precocious reduced length of telomeres, increased activity of SA-β-Gal and decreased the nuclear–cytoplasmic shuttling of SIRT1, which correspond to an activation of p53/p21 pro-senescence pathway. These results, innovatively, suggest as *Prdx6^-/-^* mice may be considered a model to study phenotypes and related processes of premature cellular aging, especially linked with metabolic disorders. Further confirming this evidence, we demonstrated as lack of Prdx6 induces sarcopenia in mice, affecting both cellular muscle differentiation and muscle atrophy mechanisms.

Previously, by using *Prdx6^-/-^* mice, we found Prdx6 is a key regulator of glucose metabolism, affecting glucose-stimulated insulin secretion, skeletal muscle insulin resistance and inflammation [8]. Aging is a natural process characterized by increased incidence of metabolic chronic diseases, such as T2DM, sharing oxidative stress and inflammation as main players [32]. Differently, T2DM has been recognized as an accelerated aging-related disease [32]. In previous studies, Prdx6 was already implicated in the regulation of aging processes. Particularly, a protective role of this enzyme against senescence was investigated on murine ocular lens (LECs), where Prdx6 is more strongly expressed compared to other Prdx family members [33]. According to previous evidences, Prdx6-deficient cells showed aberrant activation of ER stress-responsive genes/protein leading to increase in reactive oxygen species (ROS) and apoptosis [34]. On the other way, by increasing Prdx6 activity through mutant Prdx6 K122/142R (arginine), mice gained protective efficacy against oxidative stress and premature senescence in LECs [35]. Other findings demonstrated a protective role of Prdx6 in age-associated decline in mice sperm quality and fertility [36]. However, all previously discussed studies are reporting a speculative association between Prdx6 and aging without testing its direct effect on markers of senescence.

In the present study, we showed Prdx6 affects directly the cellular telomeres length by controlling the shelterin complex. This result suggests that Prdx6 plays a role in senescence-mechanism linked with genome integrity, since telomere length dynamics controls cell survival as a result of combined effect of oxidative stress, inflammation, and repeated cell replication [37]. Telomeres length decreases progressively with advancing age, and is, thus, considered a biomarker of chronological aging and age-related diseases [37]. According to this hypothesis, in *S. Cerevisiae*, mutant for the principal peroxiredoxin present in yeast, the peroxiredoxin1, also called as Tsa1, displays abnormal telomere shortening [38]. A role of Prdx6 on controlling nuclear mechanisms linked with senescence is further demonstrated by the reduction in SIRT1 nuclear localization and the correspondent activation of p53/p21 pro-aging pathway. Previous findings supported our results demonstrating as accelerated placental aging was associated with shorter telomeres, decrease in SIRT1 and increase of p53 and p21 [39]. Differently, the antioxidant action of p53 mediated by sestrins, which induces the regeneration of peroxiredoxins such as Prdx6 [40], might be not relevant or not efficacy in *Prdx6^-/-^* mice, since this antioxidant enzymes might be the major player in regulating the aging process at least in the skeletal muscle. The role of Prdx6 beyond the control of this process is highlighted by the increase in SA-β-Gal activity. Accordingly, an increase in SA-β-Gal activity was previously reported on trabecular meshwork cells knocking down for Prdx6 [11]. We moved forward by showing higher levels of SA-β-Gal activity in skeletal muscle of *Prdx6^-/-^* mice that are prone to develop diabetes [8].

As muscle is one of the principal target of insulin action and, consequently, is a major site of glucose disposal, and since muscle mass and function progressively decrease with age, the physiopathological link between sarcopenia, aging, and diabetes is so far established [3]. The insulin release deficit as well as the impaired insulin signaling leading to reduction of muscle glucose uptake present in *Prdx6^-/-^* mice [8], could explain by themselves, at least in part, the role of Prdx6 on sarcopenia. Moreover, increase in muscle and peripheral nerve oxidative damage, relevant for the onset of sarcopenia, reported in *Sod1^-/-^* mice, was associated with a specific redox shift in the catalytic Cys residue of Prdx6 (Cys47), suggesting that this process could potentially impact the development of sarcopenia during aging [41].

Nevertheless, in the present study, besides validating the role of Prdx6 in cellular aging, we further investigated its action in the pathological crosstalk between aging and diabetes mellitus by testing the mechanisms leading to muscle atrophy: protein turnover pathways, muscle differentiation, and reduction in skeletal muscle fibers. First of all, we established as absence of Prdx6 in mice resulted in a loss of muscle contractile force measured by grip test, which is commonly used to evaluate sarcopenia [42], either in forelimbs or hindlimbs. Furthermore, skeletal muscle fibers atrophy is a main pathological characteristic during the progression of sarcopenia [43]. In this study, we found a reduction in CSA of *Prdx6^-/-^* skeletal muscle fibers compared to control group, demonstrating the presence of sarcopenia. In particular, the CSA of slow twitch muscle fibers was reduced in *Prdx6^-/-^* mice. A previous study reported as subjects with T2DM showed a reduction of slow twitch fibers content [43]. These results are in agreement with a previous study in mice with impaired glucose metabolism, such as FOXO1 transgenic mice, displayed a reduced number of slow twitch muscle fibers [44]. These observations suggested that in *Prdx6^-/-^* mice the phenotype of sarcopenia might be a consequence of mild diabetes typical of these mice model. Differently, the pathophysiological process of loss in skeletal muscle mass during aging is characterized by a fast-to-slow twitch fibers shift [45]. Our results instead indicated a metabolic sarcopenia, which may be associated to a cellular specific increase in oxidative stress linked with the loss of Prdx6. Thus, the higher levels in oxidative stress may lead to metabolic dysfunction, premature aging, and associated sarcopenia (diabetic sarcopenia). Several antioxidant enzymes may be implicated in this pathological process. In particular, Prdx4 may protect from diabetes and its chronic complications, by reducing oxidative stress levels [45]. Therefore, Prdx4 potential role in diabetic sarcopenia should be investigated, as well. However, we can assert that Prdx6 is the primary antioxidant enzyme affecting the sarcopenic processes in skeletal muscle.

**Figure 7:**
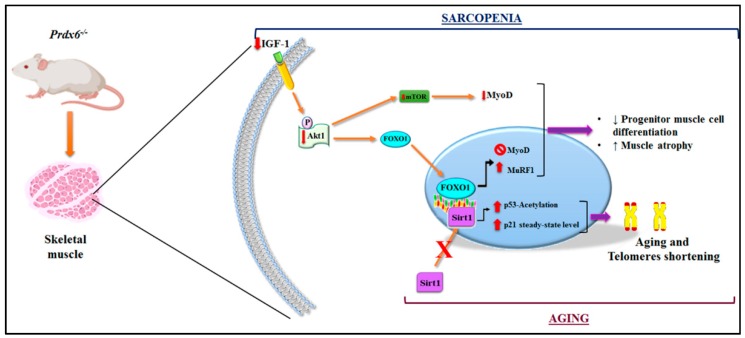
Graphical representation of Prdx6 in the regulation of mechanism related to aging and sarcopenia. In skeletal muscle of *Prdx6^-/-^* mice, the related impairment in muscle mass turnover depending on the alteration of IGF-1/Akt-1/mTOR/FOXO1 signaling pathway has been described. Moreover, the inhibition of SIRT1 nuclear translocation and activation with subsequent reduction in telomeres length and the activation of the p53/p21 pro-aging pathway has been detected. The illustration was performed by using Bio Render and Photoshop programs.

IGF-1 decline is considered a key marker on the age-related changes in body composition, especially depending on endocrine, metabolic, and nutritional statuses [46]. Therefore, IGF-1 and its downstream pathway were the main candidates in our analysis. The related impairment in skeletal muscle mass turnover was dependent on the alteration of IGF-1/Akt-1/mTOR/FOXO1 [14,47] signaling pathway in *Prdx6^-/-^* mice. This is in agreement with the inhibition in SIRT1 anti-aging pathway [39]. Moreover, it is also in line with the decline in muscle differentiation, established by the decrease of MyoD and by the reduction of the anabolic pathway, mainly mediated by mTOR, found in mice null for Prdx6. SIRT1, as well as, insulin regulatory pathways, affect muscle differentiation through modulation of several transcriptional/epigenetic co-factors, including FOXO1, which in turn regulates MyoD [48]. However, so far, these connections were not fully supported by experimental evidences and were controversial, although the role of Prdx6 on muscle differentiation via MyoD was already hypothesized [49]. Prdx6 involvement on metabolic sarcopenia is further evident by the increase in muscle atrophy markers in *Prdx6^-/-^* mice. The absence of Prdx6 by inducing a metabolic defect on muscle, linked with impairment in glycemic homeostasis and the chain of molecules that controls it, like IGF-1 [14], promotes activation of muscle catabolic pathway through the MuRF1-mediated ubiquitination of proteins. Hence, based on these results, Prdx6 may be considered a pivotal piece to make out the difficult puzzle in understanding the maintenance of muscle homeostasis (Figure 7).

Strengths of the present study include the use of well-validated knockout mice model and the stably silenced C2C12 murine myoblast cell line for Prdx6 that were innovatively proposed as model to study cellular aging and sarcopenia. Other strengths include the use of most important biomarkers for the investigated outcomes (e.g., telomeres length). The major limitations to acknowledge, even if our knockout animal model did not induce gross physiological abnormalities that can confound interpretation of our results, are on regard with the unexpected compensatory or redundancy mechanisms that might be activated when a specific gene is missing.

## 5. Conclusions

In conclusion, we demonstrated that Prdx6 plays a fundamental role in the cellular aging processes associated with DM. This occurs through the modulation of pro-aging markers and the activation of cell senescence signaling pathways. Moreover, we showed as animals lacking this antioxidant enzyme develop sarcopenia by an impairment in the main anabolic and catabolic mechanisms associated with skeletal muscle turnover. Therefore, Prdx6 may be considered as one among the principal mediators in the pathological loop between aging, oxidative stress, and DM leading to muscle loss. Further studies are necessary to clarify these complex processes. However, the use of *Prdx6^-/-^* as model of premature cellular aging/sarcopenia may help in this challenge. Together, our results suggest that Prdx6 may be proposed as a novel biomarker and pharmacological therapeutic target to prevent and cure metabolic diseases and sarcopenia associated with accelerated aging.

## Supplementary Materials

The following are available online at https://www.mdpi.com/2076-3921/9/4/329/s1, Table S1, Gene senescence’s list.
