# Supplementary material for: Prdx6 Plays a Main Role in the Crosstalk between Aging and Metabolic Sarcopenia
Source: Antioxidants (Basel). 2020 Apr 17;9(4):329. doi: 10.3390/antiox9040329 (PMC7222359; doi:10.3390/antiox9040329)
Supplement: Supplementary file 1 [file antioxidants-09-00329-s001.pdf]

Supplementary Material

Supplemental Table S1: Gene senescence's list

Abl1	Egr1	Pik3ca
Akt1	Ets1	Plau
Aldh1a3	Ets2	Prkcd
Atm	Fn1	Pten
Bmi1	Gadd45a	Rb1
Calr	Glb1	Rbl1
Ccna2	Gsk3b	Rbl2
Ccnb1	Hras1	Serpinb2
Ccnd1	Id1	Serpine1
Ccne1	Ifng	Sirt1
Cd44	Igf1	Sod1
Cdc25c	Igf1r	Sod2
Cdk2	Igfbp3	Sparc
Cdk4	Igfbp5	Tbx2
Cdk6	Igfbp7	Tbx3
Cdkn1a	Ing1	Terf2
Cdkn1b	Irf3	Tert
Cdkn1c	Irf5	Tgfb1
Cdkn2a	Irf7	Tgfb1i1
Cdkn2b	Map2k1	Thbs1
Cdkn2c	Map2k3	Trp53
Cdkn2d	Map2k6	Trp53bp1
Chek1	Mapk14	Twist1
Chek2	Mdm2	Vim
Cited2	Morc3	Actb
Col1a1	Myc	B2m
Col3a1	Nbn	Gapdh
Creg1	Nfkb1	Gusb
E2f1	Nox4	Hsp90ab1
E2f3	Pcna	
